# Risk Factors for Pancreatic Cancer in China: A Multicenter Case-Control Study

**DOI:** 10.2188/jea.JE20140148

**Published:** 2016-02-05

**Authors:** Zhaoxu Zheng, Rongshou Zheng, Yutong He, Xibin Sun, Ning Wang, Tianhui Chen, Wanqing Chen

**Affiliations:** 1 Cancer Hospital, Chinese Academy of Medical Sciences, Beijing, China; 2 National Office for Cancer Prevention and Control, National Cancer Center, Beijing, China; 3 4th affiliated Hospital of Hebei Medical University, Shi-Jia-Zhuang, Hebei, China; 4 Henan Cancer Hospital, Zhengzhou, Henan, China; 5 Beijing Office for Cancer Prevention and Control, Peking University Cancer Hospital & Institute, Beijing, China; 6 Division of Molecular Genetic Epidemiology, German Cancer Research Center, Heidelberg, Germany

**Keywords:** pancreatic cancer, multicenter, case-control study, risk factor, China

## Abstract

**Background:**

Despite having one of the highest mortality rates of all cancers, the risk factors of pancreatic cancer remain unclear. We assessed risk factors of pancreatic cancer in China.

**Methods:**

A case-control study design was conducted using data from four hospital-based cancer registries (Henan Provincial Cancer Hospital, Beijing Cancer Hospital, Hebei Provincial Cancer Hospital, and Cancer Hospital of Chinese Academy of Medical Sciences). Controls were equally matched and selected from family members of non-pancreatic cancer patients in the same hospitals. Face-to-face interviews were conducted by trained staff using questionnaires. Conditional logistic regression models were used to assess odd ratios (ORs) and 95% confident intervals (CIs).

**Results:**

Among 646 recruited participants, 323 were pancreatic cancer patients and 323 were controls. Multivariate logistic analysis suggested that pancreatic cancer family history (adjusted OR 1.23; 95% CI, 1.11–3.70), obesity (adjusted OR 1.77; 95% CI, 1.22–2.57), diabetes (adjusted OR 2.96; 95% CI, 1.48–5.92) and smoking (adjusted OR 1.78; 95% CI, 1.02–3.10) were risk factors for pancreatic cancer, but that drinking tea (adjusted OR 0.49; 95% CI, 0.25–0.84) was associated with reduced risk of pancreatic cancer.

**Conclusions:**

Cigarette smoking, family history, obesity, and diabetes are risk factors of pancreatic cancer, which is important information for designing early intervention and preventive strategies for pancreatic cancer and may be beneficial to pancreatic cancer control in China.

## INTRODUCTION

Pancreatic cancer has one of the highest mortality rates among malignancies, with an aggression behavior and a poor prognosis. Recently, pancreatic cancer has shown an increasing trend in incidence rates among both men and women.^1^^,^^2^ Approximately 266 669 cases die due to pancreatic cancer per year globally, making it the eighth leading cause of cancer death.^3^^,^^4^ Despite advances in surgery, chemotherapy, and radiotherapy, the prognosis of pancreatic cancer is still extremely poor: less than 5% of patients survive for five years after diagnosis. The crude incidence rate of pancreatic cancer in China was 7.28 cases per 100 000 people, and the incidence rate was 4.63 cases per 100 000 people after standardization using Segi’s population in 2009. The mortality rate was 6.61 deaths per 100 000 people, with significant differences in incidence and mortality rates between urban and rural areas.^5^ Although some studies have investigated the etiology of pancreatic cancer, the exact causes of pancreatic cancer remain unknown.^6^ However, some risk factors, such as lifestyle, diets, obesity, and family history of pancreatitis and diabetes, appear to be associated with pancreatic cancer.^7^^–^^13^ In this study, we investigated the main risk factors of pancreatic cancer in China, which may offer a theoretical basis for pancreatic cancer prevention.

## MATERIALS AND METHODS

### Study subjects

Our study is a hospital-based case-control study assessing the major risk factors of pancreatic cancer. High-quality hospital-based cancer registration data were selected from four hospitals: Henan Provincial Cancer Hospital, Beijing Cancer Hospital, Hebei Provincial Cancer Hospital, and the Cancer Institute & Hospital at the Chinese Academy of Medical Sciences in Beijing. A total of 646 participants were recruited, including 323 pathologically verified cancer cases and 323 controls selected from family members of other inpatients in the same hospital who did not have pancreatic cancer. Cases and control were 1:1 matched by gender and age; 110 pairs were recruited from Henan Provincial Cancer Hospital, 105 from Beijing Cancer Hospital, 73 from Hebei Cancer Hospital, and 35 from the Cancer Institute & Hospital of the Chinese Academy of Medical Sciences. The diagnoses of all pancreatic cancers were verified by histology after surgery or biopsy. Control subjects had no cancer history and were individually matched to cases with the same gender and age (within 5 years) in the same county or city. The response rate was 98% (100% for the case arm and 96% for the control arm). Ultimately, a total of 323 cases and 323 controls were recruited and analyzed in this study. All subjects gave informed consent before being interviewed. The study was approved by the ethics committees of all participating hospitals.

### Data collection

All study subjects were asked to fill out a self-administered questionnaire, which was designed by experts. The questionnaire included questions assessing cigarette smoking, alcohol drinking, tea drinking, exposure to carcinogens, environmental factors, dietary habits, family history of pancreatic disease (pancreatitis, pancreatic cyst, cholecystitis, gallstone, peptic ulcer, or cancer), and psychological factors (personal characteristics and depression). Frequent cooking was defined if subjects cooked at least once per day. Mental pressure was assessed by asking about their feelings when working and was defined as stressed, median, or relaxed. We collected detailed information on smoking, including average number of cigarettes smoked daily, smoking period (a pack-year was defined as twenty cigarettes smoked daily for one year), age at starting and quitting, and exposure to secondhand smoke. The recruitment period was between November 2011 and February 2013.

### Quality control

All cases were diagnosed by pathologists and were confirmed by attending physicians. Most questionnaires were filled out by the subjects themselves, although some (0.3%) were completed by their close relatives. The interviewers were trained on study aims, contents of the questionnaire, and interview skills. All questionnaires were cross-checked daily.

### Statistical analysis

Chi-square tests were used to assess comparison between cases and controls. Conditional logistic models were used for univariate and multivariate analysis, and multivariate analysis was adjusted for potential confounders, such as age, sex, race, and residential areas, to evaluate the association of exposure risk factors with pancreatic cancer risk. Only variables showing a significant influence in univariate analysis were included in multivariate analysis. The odd ratios (ORs) and 95% confident intervals (CIs) of the associations between risk factors and cancer risk were calculated by multivariate logistic analysis.

All analyses were performed using SPSS software (version 11.0; SPSS, Inc., Chicago, IL, USA), and a two-side *P*-value of less than 0.05 was considered to be statistically significant.

## RESULTS

There were 323 case-control pairs recruited from four hospitals (181 male pairs and 142 female pairs). Mean ages for cases were 57.37, 58.50, 59.16, and 59.31 years for Henan Provincial Cancer Hospital, Beijing Cancer Hospital, Hebei Provincial Cancer Hospital, and the Cancer Institute & Hospital of the Chinese Academy of Medical Sciences, respectively. Overall, the mean age was 58.7 years for cases and 58.0 years for controls. No significant differences between cases and controls were found for age, marital status, occupation, ethnicity, education, or residence (Table 1).

**Table 1.** Characteristics of 323 pancreatic cancer cases and 323 controls

Variables	Cases^a^		Controls^a^		X^2^	*P*
	
	*n*	%	*n*	%		
Center						
Henan Cancer Hospital	110	(34.1)	110	(34.1)		
Beijing Cancer Hospital	105	(32.5)	105	(32.5)		
Hebei Cancer Hospital	73	(22.6)	73	(22.6)		
CICAMS^b^	35	(10.8)	35	(10.8)	0.000	1.000
Sex						
Male	181	(56.0)	181	(56.0)		
Female	142	(44.0)	142	(44.0)	0.000	1.000
Age, years^c^	58.7	(11.2)	58.0	(11.2)	0.790	0.430
Ethnic group						
Han	312	(96.6)	314	(97.2)		
Other	9	(2.8)	9	(2.8)	0.000	1.000
Marital status						
Unmarried	5	(1.5)	4	(1.3)		
Married	294	(91.3)	298	(93.1)		
Divorced	7	(2.2)	2	(0.6)		
Widowed	16	(5.0)	16	(5.0)	3.248	0.517
Education, years						
<6	76	(25.0)	75	(23.7)		
6–12	111	(36.5)	118	(37.2)		
>12	117	(38.5)	124	(39.1)	0.152	0.927
Residential areas						
Urban	163	(50.6)	156	(48.9)		
Rural	159	(49.4)	163	(51.1)	0.189	0.664
Occupation						
Worker	29	(9.2)	32	(10.1)		
Peasant	117	(37.3)	117	(36.9)		
Manager	76	(24.2)	82	(25.9)		
Other	92	(29.3)	86	(27.1)	1.622	0.951

^a^Sums may not add up to total because of missing values.

^b^Cancer Institute & Hospital, Chinese Academy of Medical Sciences.

^c^Values given are mean (standard deviation).

Univariate analysis showed that cigarette smoking was associated with an increased risk of pancreatic cancer (OR 1.50; 95% CI, 1.01–2.24). Increased dose and duration of cigarette smoking were also associated with elevated risk. Those who smoked more than 30 cigarettes daily had substantially higher risk than light smokers (OR 3.67; 95% CI, 1.07–12.66 for ≥30 cigarettes versus OR 2.04; 95% CI, 1.04–4.02 for 20–29 cigarettes). Smoking more than 20 pack-years was associated with elevated risk (OR 1.48; 95% CI, 1.03–2.13) compared to nonsmokers. In univariate analyses, mental pressure (OR 1.73; 95% CI, 1.07–2.81), family history of pancreatic cancer (OR 3.67; 95% CI, 1.02–13.14), high body mass index (OR 1.67; 95% CI, 1.18–2.38), diabetes (OR 2.69; 95% CI, 1.51–4.77), gallstone (OR 2.80; 95% CI, 1.01–7.77), and habit of eating pickles (OR 3.67; 95% CI, 1.02–13.14) were associated with elevated risk, while tea (OR 0.53; 95% CI, 0.36–0.79) and vegetable (OR 0.42; 95% CI, 0.22–0.84) consumption were associated with decreased risk. After adjusting for age, sex, race, and residential areas, most factors remained associated with pancreatic cancer risk (Table 2, Table 3, and Table 4). In multivariate analyses, smoking (adjusted OR 1.78; 95% CI, 1.02–3.10), family history of pancreatic cancer (adjusted OR 1.23; 95% CI, 1.11–3.70), high body mass index (adjusted OR 1.77; 95% CI, 1.22–2.57), and diabetes (adjusted OR 2.96; 95% CI, 1.48–5.92) were associated with elevated risk. However, smoking frequency and quantities were not statistically significant in multivariate analysis. Tea drinking was associated with a 51% reduction in the risk of pancreatic cancer (adjusted OR 0.49; 95% CI, 0.25–0.84), while heavy smoking (more than 20 pack-years) nearly doubled the risk of pancreatic cancer (adjusted OR 1.78; 95% CI, 1.02–3.10) (Table 2, Table 3, and Table 4).

**Table 2.** Lifestyles associated with risk of pancreatic cancer

Risk factors	Cases		Controls		Crude OR (95% CI)	Adjusted OR1 (95% CI)^a^	Adjusted OR2 (95% CI)^b^
	
	*n*	(%)	*n*	(%)			
Smoking							
No	190	−58.8	210	−65.0	1.00 (reference)	1.00 (reference)	1.00 (reference)
Yes	133	−41.2	113	−35.0	**1.50 (1.01–2.24)**	**1.64 (1.08–2.48)**	**1.78 (1.02–3.10)**
Cigarettes per day							
<20	50	−37.6	72	−63.7	1.00 (reference)	1.00 (reference)	1.00 (reference)
20–29	61	−45.9	34	−30.1	**2.04 (1.04–4.02)**	**1.97 (1.03–4.01)**	1.55 (0.73–3.31)
≥30	22	−16.5	7	−6.2	**3.67 (1.07–12.66)**	**3.85 (1.09–13.59)**	1.94 (0.52–7.36)
Pack-years							
≤20	44	−33.1	58	−51.3	1.00 (reference)	1.00 (reference)	1.00 (reference)
>20	89	−66.9	55	−48.7	**1.48 (1.03–2.13)**	**1.45 (1.01–2.09)**	1.39 (0.86–2.23)
Alcohol consumption							
No	272	−84.2	274	−84.8	1.00 (reference)	1.00 (reference)	1.00 (reference)
Yes	51	−15.8	49	−15.2	1.07 (0.65–1.76)	1.18 (0.70–1.99)	1.22 (0.60–2.48)
Tea consumption							
No	240	−75.9	208	−64.8	1.00 (reference)	1.00 (reference)	1.00 (reference)
Yes	76	−24.1	113	−35.2	**0.53 (0.36–0.79)**	**0.55 (0.36–0.83)**	**0.49 (0.25–0.84)**
Coffee consumption							
No	308	−96.9	297	−96.4	1.00 (reference)	1.00 (reference)	1.00 (reference)
Yes	10	−3.1	11	−3.6	0.90 (0.37–2.22)	0.89 (0.33–2.37)	0.91 (0.30–2.78)
Cooking							
No	140	−44.7	130	−40.4	1.00 (reference)	1.00 (reference)	1.00 (reference)
Yes	170	−55.3	192	−59.6	0.73 (0.49–1.10)	0.69 (0.45–1.05)	0.63 (0.37–1.06)
Mental pressure							
No	265	−83.1	286	−89.1	1.00 (reference)	1.00 (reference)	1.00 (reference)
Yes	54	−16.9	35	−10.9	**1.73 (1.07–2.81)**	**1.67 (1.02–2.74)**	1.32 (0.73–2.39)

CI, confidence interval; OR, odds ratio.

All bold ORs were statistically significant as indicated by their 95% CIs.

^a^Adjusted for age, sex, race, and residential areas.

^b^Adjusted for age, sex, race, residential areas, smoking, tea drinking, mental pressure, family history of pancreatic cancer, BMI, diabetes, gallstone, pickle consumption, and vegetable consumption.

**Table 3.** Characteristics of patients associated with risk of pancreatic cancer

Characteristics	Cases		Controls		Crude OR (95% CI)	Adjusted OR1 (95% CI)^a^	Adjusted OR2 (95% CI)^b^
	
	*n*	(%)	*n*	(%)			
Family history of pancreatic cancer							
No	312	−96.6	320	−99.1	1.00 (reference)	1.00 (reference)	1.00 (reference)
Yes	11	−3.4	3	−0.9	**3.67 (1.02–13.14)**	**4.04 (1.08–15.07)**	**1.23 (1.11–3.70)**
BMI							
<24.0	197	−61.0	230	−71.2	1.00 (reference)	1.00 (reference)	1.00 (reference)
≥24.0	126	−39.0	93	−28.8	**1.67 (1.18–2.38)**	**1.78 (1.23–2.58)**	**1.77 (1.22–2.57)**
Diabetes							
No	174	−78.0	255	−90.7	1.00 (reference)	1.00 (reference)	1.00 (reference)
Yes	49	−22.0	26	−9.3	**2.69 (1.51–4.77)**	**2.60 (1.41–4.79)**	**2.96 (1.48–5.92)**
Cholecystitis							
No	272	−94.1	195	−96.7	1.00 (reference)	1.00 (reference)	1.00 (reference)
Yes	17	−5.9	10	−3.3	1.89 (0.84–4.24)	1.91 (0.83–4.38)	1.35 (0.50–3.68)
Gallstone							
No	274	−95.1	300	−98.4	1.00 (reference)	1.00 (reference)	1.00 (reference)
Yes	14	−4.9	5	−1.6	**2.80 (1.01–7.77)**	2.58 (0.89–7.44)	2.68 (0.65–11.07)

CI, confidence interval; OR, odds ratio.

All bold ORs were statistically significant as indicated by their 95% CIs.

^a^Adjusted for age, sex, race, and residential areas.

^b^Adjusted for age, sex, race, residential areas, smoking, tea drinking, mental pressure, family history of pancreatic cancer, BMI, diabetes, gallstone, pickles and vegetables.

**Table 4.** Dietary factors associated with risk of pancreatic cancer

Dietary factors	Cases		Controls		Crude OR (95% CI)	Adjusted OR1 (95% CI)^a^	Adjusted OR2 (95% CI)^b^
	
	*n*	(%)	*n*	(%)			
Pickles							
No	312	−96.6	320	−99.1	1.00 (reference)	1.00 (reference)	1.00 (reference)
Yes	11	−3.4	3	−0.9	**3.67 (1.02–13.14)**	1.59 (0.99–2.57)	0.99 (0.39–1.68)
Vegetables							
Sometimes^c^	29	−9.1	13	−4.0	1.00 (reference)	1.00 (reference)	1.00 (reference)
Always^d^	290	−90.9	309	−96.0	**0.42 (0.22–0.84)**	**0.45 (0.22–0.90)**	0.41 (0.14–1.20)
Fruits							
Sometimes^c^	144	−45.1	132	−41.0	1.00 (reference)	1.00 (reference)	1.00 (reference)
Always^d^	175	−54.9	190	−59.0	0.80 (0.57–1.14)	0.80 (0.56–1.14)	0.62 (0.37–1.04)
Fish							
Sometimes^c^	135	−42.3	134	−41.6	1.00 (reference)	1.00 (reference)	1.00 (reference)
Always^d^	184	−57.7	188	−58.4	0.97 (0.70–1.35)	0.94 (0.67–1.33)	0.59 (0.35–1.00)
Bean							
Sometimes^c^	187	−58.6	204	−63.4	1.00 (reference)	1.00 (reference)	1.00 (reference)
Always^d^	132	−41.4	118	−36.6	1.22 (0.88–1.69)	1.18 (0.84–1.65)	1.35 (0.86–2.10)

CI, confidence interval; OR, odds ratio.

All bold ORs statistically significant as indicated by their 95% CIs.

^a^Adjusted for age, sex, race, and residential areas.

^b^Adjusted for age, sex, race, residential areas, smoking, tea, mental pressure, family history of pancreatic cancer, BMI, diabetes, gallstone, pickles and vegetables.

^c^≤2 times per week.

^d^≥3 times per week.

## DISCUSSION

The etiology of pancreatic cancer is poorly understood, as many factors (genetic susceptibility, environmental factors, lifestyles and physical conditions) may contribute to its etiology. It has been reported that gender, age, and ethnicity might be associated with pancreatic cancer. Some studies suggest that smoking, unhealthy diet, obesity, and some diseases, such as pancreatitis and diabetes, are associated with elevated risk of pancreatic cancer.

Cigarette smoking, one identified risk factor for pancreatic cancer,^14^ contributes to approximately 20% of pancreatic cancer cases,^15^ and several studies have shown a dose-response relationship between cigarette smoking and elevated risk of young age at onset of pancreatic cancer.^16^ A recent meta-analysis indicated that cigarette smokers were at twice the risk of pancreatic cancer compared to never smokers, and the number of cigarettes smoked and the duration of smoking were associated with an increasing trend of elevated risk.^17^ Variable magnitudes in the effects of smoking on pancreatic cancer have been observed,^16^ which may be due to differences in smoking intensity, duration, and cumulative dose, as well as study design and sample sizes. A meta-analysis consisting of 82 cohort and case-control studies published from 1950 to 2007 reported pooled relative risks (RRs) of 1.7 (95% CI, 1.6–1.9) for current smokers and 1.2 (95% CI, 1.1–1.3) for former smokers, and the association was consistent across geographic areas by sex.^17^ In one nested case-control study^18^ including 1481 cases and 1539 controls, the ORs of pancreatic cancer were 1.1 (95% CI, 0.9–1.3) for former smokers and 1.8 (95% CI, 1.4–2.3) for current smokers compared to non-smokers. Consistent with these results, we found that there was a significantly elevated risk of pancreatic cancer for smokers and the risk was closely associated with the number of cigarettes smoked per day and pack-years of smoking. A trend of increasing risk with increasing number of cigarettes smoked was observed in univariate analysis, although the trend was not substantial in multivariate analysis (Table 2). Although further analysis of the dose-response relationship between smoking and pancreatic cancer was not possible due to the small sample size, further investigation of the influence of cigarette smoking and smoking cessation on pancreatic cancer risk is warranted.

Studies focusing on possible associations of dietary factors with cancers have reported that unhealthy diets are associated with the development of human cancers, so unhealthy diets have been postulated as risk factors for carcinogenesis.^19^ However, few studies have reported a strong relationship between dietary factors and pancreatic cancer. In our study, we found that eating pickles was associated with increased risk of pancreatic cancer; other studies have reported that pickle consumption was a risk factor for rectal cancer but not for colon cancer and was even a protection factor for stomach cancer.^20^^,^^21^ These findings indicate that the associations of dietary factors with different cancers are not consistent, suggesting that distinctive mechanisms of food components may influence cells in different tissues. Therefore, our findings should be interpreted cautiously. We found an inverse association of vegetable intake with pancreatic cancer risk, which is consistent with other studies,^22^ suggesting that vegetable consumption may be a protective factor for pancreatic cancer because vegetables contain many potentially cancer-preventive agents. These results should also be interpreted cautiously due to the lack of detailed information on intake. In addition, epidemiological data on the association of tea with pancreatic cancer are controversial. Lin et al^23^ reported that green tea consumption does not decrease the risk for pancreatic cancer in Japanese adults, a finding that is in line with the results of other studies.^24^^,^^25^ However, several studies, including the present study, have shown that increased tea consumption is associated with a reduced risk of pancreatic cancer.^26^ A recent population-based case-control study in urban Shanghai drew similar conclusion.^27^ Tea contains biologically active compounds, such as catechins, which are believed to influence cancer risk. For instance, Appari suggested that green tea-derived catechins inhibit pancreatic cancer progression by induction of miR-let7-a and by inhibition of K-ras.^28^ Our findings suggest that frequently drinking tea could decrease the risk of pancreatic cancer, but further investigation is warranted.

Family history is an identified risk factor for pancreatic cancer.^29^ Two population-based case-control studies showed that 7.8% of cases and 0.6% of controls had a family history of pancreatic cancer, and individuals who had a first-degree relative with pancreatic cancer had a 3.2-fold increased risk of pancreatic cancer (95% CI, 1.8–5.6) compared to population controls.^30^^,^^31^ Another population-based cohort study observed that individuals with one parent with pancreatic cancer had elevated risk of pancreatic cancer compared to those without a family history (standardized incidence ratio 1.73; 95% CI, 1.1–2.5),^32^ which could not be explained by environmental exposures. Another prospective cohort study also drew similar conclusions.^33^ The present study found an OR of 1.23 (95% CI, 1.11–3.70) for patients with a family history, suggesting that family history is indeed an important risk factor for pancreatic cancer. Further research is needed to determine whether the clustering of pancreatic cancer is attributable to a shared underlying genetic basis and/or environmental and other factors.

Diabetes may be a cause and also a complication of pancreatic cancer, although reports have been contradictory. Therefore, the association of diabetes with pancreatic cancer warrants further investigation. Our data suggest that diabetes is an important risk factor for pancreatic cancer (OR 2.69; 95% CI, 1.51–4.77), a result that is in line with a veterans system study reporting that diabetic patients developed pancreatic cancer more often than non-diabetic patients (hazard ratio 2.17; 95% CI, 1.70–2.77).^34^ Some studies found that new-onset diabetes had the strongest association with pancreatic cancer and was largely responsible for the link between diabetes and pancreatic adenocarcinoma, which may be due to abnormal islet cell function.^35^ An Italian study found a three-fold elevated risk of pancreatic malignancy in all patients with diabetes but no statistically significant risk for patients diagnosed ≥3 years earlier (OR 1.43; 95% CI, 0.98–2.07).^36^ A 10-year prospective study in Korea found an elevated risk of pancreatic cancer in men with longer duration of diabetes,^37^ suggesting that sex hormones may play a role. In addition, new-onset diabetes is associated with worse survival of patients with pancreatic ductal adenocarcinoma who undergo pancreatectomy^38^; conversely, diabetes mellitus, especially recent-onset diabetes, has been found to be associated with improved overall survival of pancreatic cancer patients.^39^ Whether or not diabetes is a real risk factor for pancreatic cancer remains controversial because diabetes could be a sequela of pancreatic cancer. Therefore, potential reverse causality should be cautiously considered when assessing the association of diabetes with pancreatic cancer. Moreover, many studies have reported that use of metformin (a glucose control drug used for diabetic patients) is associated with decreased risk of pancreatic malignancy and with prolonged survival, which may be due to increases in AMPK phosphorylation and inhibition of mTOR and cell growth.^40^^,^^41^

Some characteristics, such as obesity and blood pressure, which might be related with pancreatic cancer, should be considered. For instance, obesity has been identified as a risk factor for pancreatic cancer, and obese subjects have been found to have around 20% higher risk compared to normal weight individuals,^42^ though potential confounding could not be excluded. We found that obesity was associated with risk of pancreatic cancer, though other prospective studies have found that the association of obesity with pancreatic cancer varied by smoking status^43^ or gender.^44^

Our study has a number of limitations. The possibility of recall bias and selection problems cannot be completely ruled out due to the case-control study design. However, we used a multicenter design, and cases and controls were recruited from the same hospitals. We did not have information on age at onset or duration of diabetes. Additionally, dietary habits and mental depression were roughly assessed using the questionnaire. The association of body mass index with pancreatic cancer is complex and may differ across populations. Therefore, further investigation of pancreatic cancer risk factors is warranted.

In conclusion, our data suggest that family history of pancreatic cancer, obesity, diabetes, and smoking are risk factors for pancreatic cancer, while drinking tea was associated with reduced risk of pancreatic cancer.
